# Association between *ATM* rs1801516 polymorphism and cancer susceptibility: a meta-analysis involving 12,879 cases and 18,054 controls

**DOI:** 10.1186/s12885-018-4941-1

**Published:** 2018-11-01

**Authors:** Yulu Gu, Jikang Shi, Shuang Qiu, Yichun Qiao, Xin Zhang, Yi Cheng, Yawen Liu

**Affiliations:** 1 Department of Epidemiology and Biostatistics, School of Public Health, Jilin University, Changchun, 130021 China; 2 Department of Pharmacy, First Hospital of Jilin University, Changchun, 130021 China; 3 Department of Cardiovascular Center, First Hospital of Jilin University, Changchun, 130021 China

**Keywords:** ATM, rs1801516, Polymorphism, Cancer susceptibility, Meta-analysis

## Abstract

**Background:**

Ataxia telangiectasia mutated (ATM) gene plays a key role in response to DNA lesions and is related to the invasion and metastasis of malignancy. Epidemiological studies have indicated associations between *ATM* rs1801516 polymorphism and different types of cancer, but their results are inconsistent. To further evaluate the effect of *ATM* rs1801516 polymorphism on cancer risk, we conducted this meta-analysis.

**Methods:**

Studies were identified according to specific inclusion criteria by searching PubMed, Web of Science, and Embase databases. Pooled odds ratios (*OR*s) and corresponding 95% confidence intervals (*CI*s) under recessive, dominant, codominant, and overdominant models of inheritance were calculated to estimate the association between rs1801516 polymorphism and cancer risk.

**Results:**

A total of 37 studies with 12,879 cases and 18,054 controls were included in our study. No significant association was found between rs1801516 polymorphism and cancer risk in overall comparisons (AA vs GG + GA: *OR* = 0.91, 95% *CI*, 0.78–1.07; AA+GA vs GG: *OR* = 1.00, 95% *CI*, 0.90–1.11; AA vs GG: *OR* = 0.89, 95% *CI*, 0.75–1.06; GA vs GG: *OR* = 1.01, 95% *CI*, 0.91–1.13; GG + AA vs GA: *OR* = 1.00, 95% *CI*, 0.88–1.10). However, after subgroup analyses by region-specified population, significant associations were found in European (AA vs GG + GA: *OR* = 0.79, 95% *CI*, 0.65–0.96, *P* = 0.017; AA vs GG: *OR* = 0.79, 95% *CI*, 0.65–0.96, *P* = 0.017), South American (AA+GA vs GG: *OR* = 2.15, 95% *CI*, 1.37–3.38, *P* = 0.001; GA vs GG: *OR* = 2.19, 95% *CI*, 1.38–3.47, *P* = 0.001; GG + AA vs GA: *OR* = 0.46, 95% *CI*, 0.29–0.72, *P* = 0.001), and Asian (AA vs GG + GA: *OR* = 7.45, 95% *CI*, 1.31–42.46, *P* = 0.024; AA vs GG: *OR* = 7.40, 95% *CI*, 1.30–42.19, *P* = 0.024). Subgroup analyses also revealed that compared with subjects carrying a GG genotype, those carrying a homozygote AA had a decreased risk for breast cancer (AA vs GG: *OR* = 0.76, 95% *CI*, 0.59–0.98, *P* = 0.035), and the homozygote AA was associated with decreased cancer risk in subjects with family history (AA vs GG: *OR* = 0.68, 95% *CI*, 0.47–0.98, *P* = 0.039).

**Conclusions:**

*ATM* rs1801516 polymorphism is not associated with overall cancer risk in total population. However, for subgroup analyses, this polymorphism is especially associated with breast cancer risk; in addition, it is associated with overall cancer risk in Europeans, South Americans, Asians, and those with family history.

**Electronic supplementary material:**

The online version of this article (10.1186/s12885-018-4941-1) contains supplementary material, which is available to authorized users.

## Background

Cancer is a worldwide public health problem, and considerable parts of death are due to cancer every year. It is reported that one fourth deaths in the United States is caused by cancer [1]. According to the latest cancer data from the GLOBOCAN website, there were 14.1 million new cancer cases, 8.2 million cancer deaths, and 32.6 million people living with cancer (within 5 years of diagnosis) in 2012 worldwide [2]. The statistical data of cancer in 2017 shows that 1,688,780 new cancer cases (836,150 males and 852,630 females) are expected to be diagnosed in the United States, and 600,920 Americans (318,420 males and 282,500 females) are expected to die of cancer [3]. For all sites combined, both the incidence rate and death rate are higher in males than those in females, and the most commonly diagnosed cancers are lung cancer, prostate cancer, breast cancer, and colon cancer [3].

Pathogenesis of cancer has been studied worldwide for a long time, generating different theories, such as the gene mutation, oxidative stress, and ionization radiation theories. Single nucleotide polymorphisms (SNPs) on different genes have been detected for finding specific biomarkers in different cancers. Ataxia telangiectasia-mutated (ATM) gene is one of the most frequently studied genes in cancer occurrence and progression. Mutation on *ATM* leads to the human autosomal recessive disorder, ataxia-telangiectasia (A-T), resulting in high cellular radiosensitivity, chromosomal instability, immunodeficiency, and cancer predisposition [4, 5]. Lymphomas and leukemia are predominant in all types of cancer in A-T patients, and the cancer incidence rate in black A-T patients is as more than two times as that in whites [6, 7]. ATM gene is located in human chromosome 11q22–23, spans over 160 kb DNA, and encodes a 315 kDa protein. As a member belonging to the phosphoinositide 3-kinase (PI3-K)-related protein kinase family, ATM is activated by a series of cellular stress events, such as DNA double-strand break (DSB), reactive oxygen species, hypotonic stress, and chloroquine [8]. ATM is involved in important life processes, including DNA repair, cell cycle regulation, neuroprotection, immunity, metabolism, longevity, and fertility [8].

Several *ATM* polymorphism loci have been studied in different types of cancer, including rs1801516, which is a common nonsynonymous variant on this gene. Genome-wide association studies (GWAS) have identified rs1801516 as a susceptibility locus for melanoma [9]. Large-sample case-control studies have assessed effects of this polymorphism on risk of breast cancer, prostate cancer, rectal cancer, bladder cancer, lung cancer, pancreatic cancer, and thyroid cancer. Meta-analyses have also been performed to assess *ATM* rs1801516 polymorphism and cancer predisposition, but the results are inconsistent [10–14].

We performed this meta-analysis to further identify the association between rs1801516 polymorphism and cancer risk using larger sample size than ever before, and using the trial sequential analysis (TSA) to give more comprehensive conclusions.

## Methods

We conducted this meta-analysis according to the Preferred Reporting Items for Systematic Reviews and Meta-Analyses (PRISMA) guidelines [15].

### Search strategy

Systematic search of publications was performed in PubMed, Web of Science, and Embase datasets (last search on November 18, 2017). Because of different nomenclatures for SNP, we took all the names that might be used in different studies of this SNP into consideration in our searching terms: “(rs1801516 or G5557A or 5557G>A or 5557 G/A or Asp1853Asn or D1853N or G1853A) and (cancer or carcinoma or malignancy)”.

### Inclusion criteria

Studies included in this meta-analysis met the following criteria: (1) A human study with full text available; (2) A study on *ATM* rs1801516 polymorphism and cancer risk; (3) Using a case-control study design; (4) Using healthy subjects without malignant diseases as controls; (5) Genotype data is sufficient for odds ratio (*OR*) and 95% confidence interval (*CI*) estimation. In addition, we screened the reference lists of all the relevant studies, including eligible studies, reviews and meta-analyses, and only original articles published in English were included.

### Data extraction

For each included study, the following information was extracted: the first author, year of publication, country, region-specified population, cancer type, source of controls, matching criteria of controls, family history, genotyping method, Hardy-Weinberg equilibrium (HWE) in controls, minor allele frequency (MAF) in cases and controls, sample size, and numbers of cases and controls with different genotype. Region-specified population in our meta-analysis was defined geographically as European, North American, South American, Asian, and Oceanian. Population-based controls (PBC) and hospital-based controls (HBC) were classified in our meta-analysis: blood donors and controls recruited from birth cohort, general population, and community are defined as PBC; and controls recruited from hospitals, clinics, research institutions, and biorepository were defined as HBC.

### Quality assessment

Two authors (YG and JS) assessed the quality of each study independently according to the Newcastle-Ottawa Scale (NOS) for case-control studies [16]. A study can be awarded a maximum score of 9: 4 assigned for selection, 2 for comparability, and 3 for exposure. When inconsistency existed between the two authors, the third author (SQ) was requested to reassess the score of quality.

### Statistical analysis

Allele and genotype frequencies in controls were calculated for each study to evaluate the HWE using chi-square test. Association between rs1801516 polymorphism and cancer risk was assessed by *OR* and corresponding 95% *CI* calculated from logistic regression. For each analysis, stratified or pooled, five comparisons were conducted, including dominant model (GA/AA vs GG), codominant model (GA vs GG and AA vs GG), recessive (AA vs GG/GA), and overdominant model (GA vs GG/AA). For studies of Sommer SS et al. [17], Gonzalez-Hormazabal P et al. [18], Maillard S et al. [19], and Calderon-Zuniga Fdel C et al. [20], no AA genotype was detected in either case or control group; thus, these studies were excluded in comparisons of AA vs GG and AA vs GA/GG. For studies of Yang H et al. [21], Bretsky P et al. [22], and Hirsch AE et al. [23], frequencies of GG and GA genotypes were presented together as GG/GA; thus, only association under recessive model was evaluated for these studies. For study of Xu L et al. [24], frequencies of GA and AA genotypes were presented together as GA/AA; thus, only association under dominant model was evaluated for this study. Subgroup analyses were performed by cancer type, region-specified population, source of control, matching status of controls, family history, sample size, and HWE in controls. Heterogeneity among studies was evaluated using *Q* test and *I*^*2*^ statistics. Fixed effect model (Mantel-Haenszel method) was used to calculate *OR* and 95% *CI* when *P* value of *Q* test was more than 0.10 or *I*^*2*^ value was less than 50%; otherwise, random effect model (DerSimonian-Laird method) was used. When the meta-analysis included 10 studies or more, publication bias was estimated using the visualizing Begg’s funnel plot, in which the *log(OR)* and its standard error of each study were indicated as Y- and X- axes respectively. An asymmetric funnel plot implied a possible publication bias. Furthermore, Egger’s linear regression test was utilized to determine the significance of asymmetry (*P* < 0.05 was considered to represent significant publication bias). Sensitivity analysis was performed with one study omitted at each time.

All analyses were performed using Stata 12.0, and two-sided tests with *P* value less than 0.05 was considered statistically significant unless otherwise specified.

### Trial sequential analysis

Because of sparse data and repeated significance testing, meta-analyses may lead to type I error for the presence of systematic errors (bias) or random errors (play of chance) [25–27]. To assess our meta-analysis comprehensively, we performed TSA using the novel TSA software [28] to calculate the required information size (sample size) with an adjusted significance level. Briefly, we calculated the required information size on the basis of an overall type I error of 5%, an overall type II error of 20% (a power of 80%), and a relative risk reduction of 20%. Two-sided graphs were plotted using dotted black lines indicating boundaries for significance in a conventional meta-analysis, blue line indicating the cumulative *Z*-score, and red lines sloping inwards indicating trial sequential monitoring boundaries using adjusted *P* values.

## Results

### Study characteristics

**Fig. 1:**
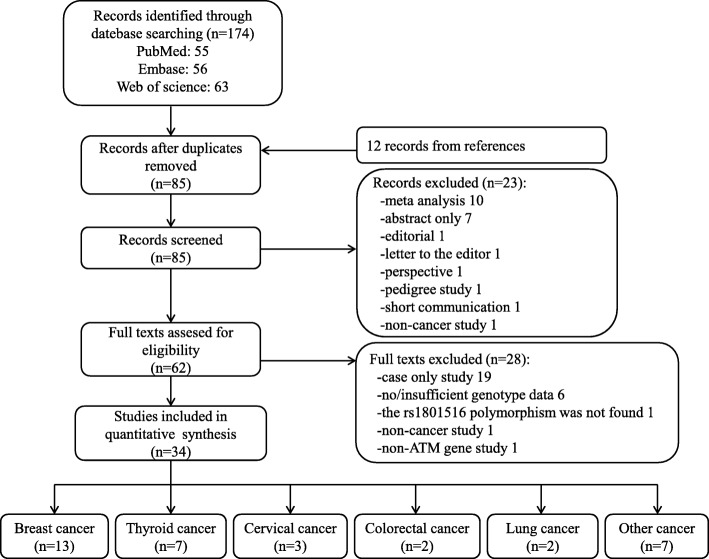
Flow chart of the process of study identification and selection

After strict screening, 34 eligible studies with 12,879 cases and 18,054 controls were identified in our meta-analysis (Fig. 1). In studies of Xu L et al. [24], Tommiska J et al. [29], and Akulevich NM et al. [30], two independent case-control studies were presented respectively; thus, each study was treated separately in our meta-analysis. For study of Xu L et al. [24], two parts of controls (HBC and PBC) were included; for study of Tommiska J et al. [29], two parts of cases (familial and unselected cases) were included; for study of Akulevich NM et al. [30], based on the condition of ionizing radiation (IR)-exposed or not, two separate studies were included, namely IR-induced papillary thyroid cancers (PTCs) vs IR-exposed controls, and sporadic PTCs vs non-exposed controls. Finally, 37 studies were included in the following analyses: 14 studies concentrated on effect of rs1801516 polymorphism on breast cancer risk [17, 18, 20, 22, 23, 29, 31–37], nine on thyroid cancer risk [19, 24, 30, 38–41], three on cervical cancer risk [42–44], two on colorectal cancer risk [45, 46], two on lung cancer risk [21, 47], one on bladder cancer risk [48], one on head and neck cancer risk [49], one on malignant melanoma risk [50], one on ovarian cancer risk [51], one on pancreatic cancer risk [52], one on prostate cancer risk [53], and one on renal cell cancer risk [54], respectively. Main characteristics of these studies are shown in Table 1 and Additional file 1: Table S1. Region-specified population was defined geographically in the 37 studies, 19 of which was European, 12 of which was North American, two of which was South American, three of which was Asian, and one of which was Oceanian. Cases in seven studies had a family history, and cases in the other 30 studies were unselected. Controls in 14 studies were HBC, controls in 17 studies were PBC, and six studies didn’t report the source of controls. A total of 25 studies had controls matched to cases for different factors; whereas, 12 studies had controls not matched to cases in that the controls were randomly selected. Genotyping methods were diverse, including real time polymerase chain reaction (RT-PCR), PCR-restriction fragment length polymorphism (PCR-RFLP), TaqManSNP (TaqMan), direct sequencing, microarray, and ten other methods. NOS scores of the included studies ranged from six to nine, indicating that the quality of studies in our meta-analysis is high.

**Table 1:** Main characteristics of the eligible studies included in the meta-analysis

First author	Year	Country	Region-specified population	Cancer type	Source of controls	Matched controls	Family history	N		NOS Score
								Cases	Controls	
Maillet P [45]	2000	Swiss	European	Colorectal cancer	PBC	No	Yes	47	163	7
Dork T [34]	2001	Germany	European	Breast cancer	PBC	Yes	No	1000	500	8
Sommer SS [17]	2002	USA	North American	Breast cancer	HBC	Yes	No	43	43	7
Angele S [33]	2003	France	European	Breast cancer	PBC	Yes	No	254	312	8
Bretsky P [22]	2003	USA	North American	Breast cancer	PBC	Yes	No	428	426	9
Angele S [53]	2004	UK	European	Prostate cancer	PBC	No	No	637	445	7
Buchholz TA [37]	2004	USA	North American	Breast cancer	PBC	No	No	58	528	7
Kristensen AT [46]	2004	Norway	European	Colorectal cancer	PBC	No	No	151	3526	7
Heikkinen K [35]	2005	Finland	European	Breast cancer	PBC	Yes	Yes	121	306	9
Landi S [47]	2006	Six countries^c^	European	Lung cancer	HBC	Yes	No	299	317	8
Renwick A [36]	2006	UK	European	Breast cancer	PBC	No	Yes	443	521	7
Tommiska J ^a^ [29]	2006	Finland	European	Breast cancer	NR	Yes	Yes	786	708	7
Tommiska J ^b^ [29]	2006	Finland	European	Breast cancer	NR	Yes	No	884	708	7
Wu X [48]	2006	USA	North American	Bladder cancer	HBC	Yes	No	696	629	8
Yang H [21]	2007	USA	North American	Lung cancer	HBC	Yes	No	556	556	8
Gonzalez-Hormazabal P [18]	2008	Chile	South America	Breast cancer	HBC	Yes	Yes	126	200	8
Hirsch AE [23]	2008	USA	North American	Breast cancer	HBC	Yes	No	37	95	7
Margulis V [54]	2008	USA	North American	Renal cell cancer	PBC	Yes	No	326	335	9
Schrauder M [32]	2008	Germany	European	Breast cancer	HBC	Yes	No	514	511	8
Tapia T [31]	2008	Chile	South America	Breast cancer	PBC	No	Yes	95	200	7
Akulevich NM ^a^ [30]	2009	Russian, Belarus	European	Thyroid cancer	PBC	Yes	No	123	198	9
Akulevich NM ^b^ [30]	2009	Russia	European	Thyroid cancer	PBC	Yes	No	132	398	9
Li D [52]	2009	USA	North American	Pancreatic cancer	HBC	Yes	No	734	780	8
Oliveira S [43]	2011	Portuguese	European	Cervical cancer	HBC	No	No	149	280	6
Al-Hadyan KS [49]	2012	Saudi Arabia	Asian	Head and neck cancer	NR	No	No	156	251	6
Xu L ^a^ [24]	2012	USA	North American	Thyroid cancer	HBC	No	No	303	511	6
Xu L ^b^ [24]	2012	USA	North American	Thyroid cancer	PBC	Yes	No	289	374	9
Alsbeih G [42]	2013	Saudi Arabia	Asian	Cervical cancer	NR	Yes	No	100	100	8
Pena-Chilet M [50]	2013	Spanish	European	Malignant melanoma	HBC	Yes	No	566	347	7
Calderon-Zuniga Fdel C [20]	2014	Mexico	North American	Breast cancer	HBC	No	Yes	94	96	6
Damiola F [39]	2014	Belarus	European	Thyroid cancer	PBC	Yes	No	83	324	9
Wojcicka A [38]	2014	Poland	European	Thyroid cancer	HBC	No	No	1603	1844	6
Maillard S [19]	2015	France	Oceanian	Thyroid cancer	PBC	Yes	No	177	275	9
Pereda CM [41]	2015	Cuba	North American	Thyroid cancer	PBC	Yes	No	203	212	9
Tecza K [51]	2015	Poland	European	Ovarian cancer	HBC	Yes	No	225	348	7
Halkova T [40]	2016	Czech Republic	European	Thyroid cancer	NR	No	No	209	374	6
Al-Harbi NM [44]	2017	Saudi Arabia	Asian	Cervical cancer	NR	Yes	No	232	313	7

*HBC*, hospital-based case–controls; *PBC,* population-based case–controls; *NR*, not report

^a,b^ Two independent case-control studies were presented for the same original study

^c^This study was conducted in six Central and Eastern European countries: Czech Republic, Hungary, Poland, Romania, Russia, and Slovakia

HWE in controls and MAF in cases and controls for each study were obtained after reading the full text or calculated according to the genotype data (Table 2). As a result, rs1801516 genotype distribution of controls was in HWE for 30 studies, and was not in HWE for four studies; besides, genotype distribution could not be obtained for three studies. Therefore, to assess the potential influence of HWE on the overall results, subgroup analysis by HWE in controls was performed. For study of Calderon-Zuniga Fdel C et al., the minor allele A was not detected in controls.

**Table 2:** Genotype distribution in cases and controls of the eligible studies

First author	Year	Cases			Controls			HWE in controls	MAF (cases/controls)
		GG	GA	AA	GG	GA	AA		
Maillet P [45]	2000	34	13	0	120	40	3	0.874	0.138 / 0.141
Dork T [34]	2001	753	235	12	422	74	4	0.705	0.130 / 0.082
Sommer SS [17]	2002	38	5	0	32	11	0	0.336	0.058 / 0.128
Angele S [33]	2003	240	65	7	192	56	6	0.433	0.127 / 0.134
Bretsky P [22]	2003	335^c^		47	329^c^		47	NA	NA
Angele S [53]	2004	457	153	18	309	124	12	0.917	0.150 / 0.166
Buchholz TA [37]	2004	39	17	2	394	119	15	0.107	0.181 / 0.141
Kristensen AT [46]	2004	99	50	2	2413	1008	105	0.983	0.179 / 0.173
Heikkinen K [35]	2005	68	44	9	174	109	23	0.308	0.256 / 0.253
Landi S [47]	2006	205	73	7	238	63	3	0.602	0.153 / 0.113
Renwick A [36]	2006	339	98	6	371	131	19	0.088	0.124 / 0.162
Tommiska J ^a^ [29]	2006	485	285	33	404	260	38	0.648	0.219 / 0.239
Tommiska J ^b^ [29]	2006	469	276	33	404	260	38	0.648	0.220 / 0.239
Wu X [48]	2006	434	156	18	439	136	17	0.109	0.158 / 0.144
Yang H [21]	2007	537^c^		7	536^c^		10	0.590	NA
Gonzalez-Hormazabal P [18]	2008	100	26	0	174	26	0	0.326	0.103 / 0.065
Hirsch AE [23]	2008	29^c^		8	78^c^		17	NA	NA
Margulis V [54]	2008	254	64	5	249	81	5	0.583	0.115 / 0.136
Schrauder M [32]	2008	406	99	9	369	129	13	0.668	0.114 / 0.152
Tapia T [31]	2008	74	19	1	183	15	2	0.015	0.112 / 0.048
Akulevich NM ^a^ [30]	2009	95	25	2	138	53	7	0.501	0.119 / 0.169
Akulevich NM ^b^ [30]	2009	105	24	3	293	90	15	0.020	0.114 / 0.151
Li D [52]	2009	524	186	18	565	200	8	0.034	0.152 / 0.140
Oliveira S [43]	2011	113	31	5	194	79	7	0.755	0.138 / 0.166
Al-Hadyan KS [49]	2012	131	23	2	218	33	0	0.265	0.087 / 0.066
Xu L ^a^ [24]	2012	239	64^d^		392	119^d^		> 0.05	NA
Xu L ^b^ [24]	2012	244	45^d^		305	69^d^		> 0.05	NA
Alsbeih G [42]	2013	90	8	2	88	12	0	0.523	0.060 / 0.060
Pena-Chilet M [50]	2013	349	91	9	232	68	11	0.040	0.121 / 0.145
Calderon-Zuniga Fdel C [20]	2014	82	12	0	96	0	0	NA	0.060 / 0.000
Damiola F [39]	2014	63	6	1	177	66	7	0.778	0.057 / 0.160
Wojcicka A [38]	2014	1261	319	23	1455	357	32	0.066	0.114 / 0.114
Maillard S [19]	2015	164	11	0	262	8	0	0.805	0.031 / 0.015
Pereda CM [41]	2015	153	44	0	162	42	2	0.690	0.112 / 0.112
Tecza K [51]	2015	153	64	6	254	76	5	0.800	0.170 / 0.128
Halkova T [40]	2016	158	45	5	284	81	9	0.270	0.132 / 0.132
Al-Harbi NM [44]	2017	201	28	3	275	38	0	0.253	0.073 / 0.061

*HWE*, hardy-weinberg equilibrium*; MAF*, minor allele frequency; *NA*, data was unavailable

^a,b^Two independent case-control studies were presented for the same original study

^c^number of GG + GA

^d^number of GA + AA

### Main results of meta-analyses

The pooled and subgroup meta-analyses of associations between rs1801516 polymorphism and cancer susceptibility are shown in Table 3. Overall, no significant association was found under any model of inheritance (AA vs GG + GA: *OR* = 0.91, 95% *CI*, 0.78–1.07; AA+GA vs GG: *OR* = 1.00, 95% *CI*, 0.90–1.11; AA vs GG: *OR* = 0.89, 95% *CI*, 0.75–1.06; GA vs GG: *OR* = 1.01, 95% *CI*, 0.91–1.13; GG + AA vs GA: *OR* = 1.00, 95% *CI*, 0.88–1.10). In subgroup analyses by region-specified population, significant associations were found in European (AA vs GG + GA: *OR* = 0.79, 95% *CI*, 0.65–0.96, *P* = 0.017; AA vs GG: *OR* = 0.79, 95% *CI*, 0.65–0.96, *P* = 0.017), South American (AA+GA vs GG: *OR* = 2.15, 95% *CI*, 1.37–3.38, *P* = 0.001; GA vs GG: *OR* = 2.19, 95% *CI*, 1.38–3.47, *P* = 0.001; GG + AA vs GA: *OR* = 0.46, 95% *CI*, 0.29–0.72, *P* = 0.001), and Asian (AA vs GG + GA: *OR* = 7.45, 95% *CI*, 1.31–42.46, *P* = 0.024; AA vs GG: *OR* = 7.40, 95% *CI*, 1.30–42.19, *P* = 0.024). In subgroup analyses by cancer types, significant decreased risk of breast cancer was found for those carrying AA genotype (AA vs GG: *OR* = 0.76, 95% *CI*, 0.59–0.98, *P* = 0.035). In subgroup analyses by family history, AA carriers had a significant decreased risk compared with GG carriers in those with family history (AA vs GG: *OR* = 0.68, 95% *CI*, 0.47–0.98, *P* = 0.039), and a borderline significance was found for AA vs GG + GA (*OR* = 0.70, 95% *CI*, 0.48–1.00, *P* = 0.051).

**Table 3:** Overall and subgroup meta-analyses of association between rs1801516 polymorphism and cancer susceptibility under different models

Group	AA vs GG + GA			AA+GA vs GG			AA vs GG			GA vs GG			GG + AA vs GA		
	*OR* (95% CI)	*I*^*2*^ (%)	*P*_h_	*OR* (95% CI)	*I*^*2*^ (%)	*P*_h_	*OR* (95% CI)	*I*^*2*^ (%)	*P*_h_	*OR* (95% CI)	*I*^*2*^ (%)	*P*_h_	*OR* (95% CI)	*I*^*2*^ (%)	*P*_h_
**Overall**	0.91 (0.78, 1.07)	< 0.1	0.616	1.00 (0.90, 1.11)	60.8	< 0.001	0.89 (0.75, 1.06)	5.8	0.378	1.01 (0.91, 1.13)	77.14	< 0.001	1.00 (0.88, 1.10)	74.4	< 0.001
**Region-specified population**															
European	**0.79 (0.65, 0.96)**	< 0.1	0.848	0.94 (0.82, 1.07)	65.6	< 0.001	**0.79 (0.65, 0.96)**	< 0.1	0.677	0.95 (0.84, 1.08)	61.9	< 0.001	1.04 (0.92, 1.18)	60.0	< 0.001
North American	1.09 (0.82, 1.45)	< 0.1	0.566	1.02 (0.90, 1.15)	43.6	0.077	1.32 (0.85, 2.06)	< 0.1	0.408	1.03 (0.80, 1.32)	52.3	0.050	0.97 (0.76, 1.25)	52.5	0.049
South American	1.07 (0.1, 11.88)	–	–	**2.15 (1.37, 3.38)**	16.6	0.274	1.24 (0.11, 13.84)	–	–	**2.19 (1.38, 3.47)**	33.2	0.221	**0.46 (0.29, 0.72)**	32.7	0.223
Asian	**7.45 (1.31, 42.46)**	< 0.1	0.956	1.11 (0.79, 1.58)	< 0.1	0.719	**7.40 (1.30, 42.19)**	< 0.1	0.949	1.00 (0.70, 1.43)	< 0.1	0.592	1.02 (0.71, 1.46)	< 0.1	0.584
Oceanian	1.83 (0.55, 6.05)	–	–	2.20 (0.87, 5.58)	–	–	–	–	–	2.20 (0.87, 5.58)	–	–	0.46 (0.18, 1.16)	–	–
**Cancer type**															
Breast cancer	0.84 (0.68, 1.04)	< 0.1	0.777	1.09 (0.86, 1.38)	78.0	< 0.001	**0.76 (0.59, 0.98)**	< 0.1	0.632	1.11 (0.87, 1.41)	76.4	< 0.001	0.90 (0.71, 1.13)	75.3	< 0.001
Thyroid cancer	0.73 (0.48, 1.11)	< 0.1	0.886	0.87 (0.71, 1.07)	52.3	0.033	0.71 (0.47, 1.08)	< 0.1	0.830	0.89 (0.67, 1.18)	60.6	0.018	1.11 (0.84, 1.47)	59.1	0.023
Cervical cancer	2.29 (0.89, 5.91)	< 0.1	0.379	0.86 (0.63, 1.18)	< 0.1	0.443	2.13 (0.83, 5.48)	5.8	0.346	0.79 (0.57, 1.09)	< 0.1	0.489	1.29 (0.93, 1.79)	< 0.1	0.494
Colorectal cancer	0.44 (0.12, 1.59)	< 0.1	0.953	1.13 (0.83, 1.54)	< 0.1	0.874	0.47 (0.13, 1.69)	< 0.1	0.966	1.20 (0.87, 1.64)	< 0.1	0.899	0.82 (0.60, 1.12)	< 0.1	0.903
Lung cancer	1.22 (0.35, 4.20)	55.4	0.134	1.41 (0.97, 2.05)	–	–	2.71 (0.69, 10.61)	–	–	1.35 (0.92, 1.98)	–	–	0.76 (0.52, 1.12)	–	–
**Source of controls**															
PBC	0.83 (0.65, 1.07)	< 0.1	0.858	0.99 (0.81, 1.21)	70.8	< 0.001	0.75 (0.55, 1.03)	< 0.1	0.771	1.03 (0.83, 1.28)	70.8	< 0.001	0.96 (0.78, 1.19)	69.9	< 0.001
HBC	1.04 (0.80, 1.36)	17.2	0.285	1.02 (0.86, 1.20)	62.5	0.002	1.06 (0.80, 1.42)	36.2	0.140	1.03 (0.86, 1.23)	62.2	0.003	0.98 (0.82, 1.17)	60.8	0.004
NR	0.88 (0.64, 1.20)	24.0	0.254	0.93 (0.82, 1.06)	< 0.1	0.827	0.86 (0.63, 1.18)	26.2	0.238	0.93 (0.82, 1.07)	< 0.1	0.928	1.06 (0.93, 1.20)	< 0.1	0.944
**Matched controls**															
Yes	0.95 (0.79, 1.14)	< 0.1	0.514	0.97 (0.84, 1.12)	64.8	< 0.001	0.93 (0.75, 1.15)	18	0.238	0.99 (0.86, 1.14)	62.4	< 0.001	1.01 (0.88, 1.16)	60.5	< 0.001
No	0.82 (0.59, 1.12)	< 0.1	0.567	1.02 (0.86, 1.21)	54.9	0.011	0.81 (0.59, 1.11)	< 0.1	0.565	1.06 (0.87, 1.29)	58.2	0.008	0.94 (0.77, 1.14)	57.9	0.008
**Family history**															
Yes	0.70 (0.48, 1.00)	< 0.1	0.574	1.20 (0.85, 1.71)	73.1	0.001	**0.68 (0.47, 0.98)**	< 0.1	0.519	1.25 (0.88, 1.77)	71.2	0.002	0.80 (0.57, 1.12)	69.9	0.003
No	0.98 (0.82, 1.16)	< 0.1	0.634	0.97 (0.87, 1.09)	57.9	< 0.001	0.97 (0.79, 1.18)	5.2	0.39	0.98 (0.87, 1.11)	57.5	< 0.001	1.02 (0.90, 1.14)	56	< 0.001
**Sample size**															
< 1000	0.95 (0.75, 1.20)	< 0.1	0.601	0.99 (0.85, 1.15)	56.8	< 0.001	0.89 (0.67, 1.20)	4.7	0.399	1.01 (0.85, 1.19)	56.4	< 0.001	0.99 (0.84, 1.17)	54.9	0.001
> 1000	0.89 (0.72, 1.10)	2.0	0.420	1.01 (0.87, 1.17)	71.6	< 0.001	0.89 (0.71, 1.11)	18.1	0.282	1.02 (0.88, 1.18)	69.9	0.001	0.98 (0.84, 1.13)	68.7	0.001
**HWE in controls**															
Yes	0.87 (0.73, 1.05)	< 0.1	0.74	0.99 (0.89, 1.10)	57.5	< 0.001	0.87 (0.72, 1.05)	< 0.1	0.58	1.00 (0.89, 1.12)	56.7	< 0.001	0.99 (0.89, 1.11)	54.9	< 0.001
No	1.09 (0.64, 1.84)	56.0	0.078	1.06 (0.70, 1.61)	76.3	0.005	0.95 (0.36, 2.50)	58.3	0.066	1.08 (0.72, 1.63)	74.4	0.008	0.92 (0.62, 1.38)	73.5	0.010
NR	1.03 (0.69, 1.52)	< 0.1	0.631	29.24 (1.71, 501.48)	–	–	–	–	–	29.24 (1.71, 501.48)	–	–	**0.03 (0.00, 0.59)**	–	–

*HBC*, hospital-based case–controls; *PBC*, population-based case–controls; *NR*, not report

*P*_h_, *P* value of *Q* test for heterogeneity

Significant *OR*s (95% CIs) were in bold

### Heterogeneity analysis

We applied *Q* test and *I*^*2*^ statistics to evaluate the heterogeneity of our meta-analysis. Our results showed significant heterogeneity among studies for AA+GA vs GG (*I*^*2*^ = 60.8%, *P* < 0.001), GA vs GG (*I*^*2*^ = 77.1%, *P* < 0.001), and GG + AA vs GA (*I*^*2*^ = 74.4%, *P* < 0.001) models (Table 3). To further investigate the source of heterogeneity, we performed meta-regression analysis by region-specified population, cancer type, source of controls, matched controls or not, family history, sample size, and HWE in controls. As a result, family history was a source of heterogeneity for AA+GA vs GG (*P* = 0.040, 59% *CI*, 0.204–7.804) and GA vs GG (*P* = 0.044, 59% *CI*, 0.113–8.055), suggesting that family history may explain the among-studies’ heterogeneity under these two models. However, no factor was detected as a source of heterogeneity for GG + AA vs GA (Table 4).

**Table 4:** The meta-regression results of the association between the rs1801516 polymorphism and cancer risk

Comparisons	Coef.	Std. Err.	*t*	*P*	95% *CI*	*τ* ^2^	*I*^2^ res (%)	Adj *R*^2^ (%)	F	*P*_J_
AA+GA vs GG										
Region-specified population						0.209	93.570	−7.280	0.580	0.676
European	−1.245	4.907	−0.250	0.801	(−11.280, 8.790)					
North American	1.646	5.043	0.330	0.747	(−8.668, 11.959)					
South American	0.128	5.853	0.020	0.983	(−11.843, 12.098)					
Asian	−1.132	5.520	− 0.210	0.839	(−12.421, 10.157)					
Oceanian	referent	referent	referent	referent	referent					
Cancer type						0.221	94.150	−13.690	0.360	0.870
Breast cancer	2.273	3.744	0.610	0.549	(−5.396, 9.942)					
Cervical cancer	−0.220	4.474	− 0.050	0.961	(−9.383, 8.944)					
Lung cancer	0.304	5.996	0.050	0.960	(−11.979, 12.587)					
Thyroid cancer	−0.147	3.830	− 0.040	0.970	(−7.993, 7.698)					
Other cancer	−0.049	3.927	−0.010	0.990	(−8.093, 7.996)					
Colorectal cancer	referent	referent	referent	referent	referent					
Source of controls	2.066	1.965	1.050	0.303	(−1.974, 6.106)	0.246	94.590	0.040		
Matched controls	−2.282	1.640	−1.390	0.174	(−5.624, 1.059)	0.189	93.410	2.740		
Family history	4.0038	1.866	2.150	**0.040**	**(0.204, 7.804)**	0.174	93.400	10.750		
Sample size	−1.069	1.8193	−0.59	0.561	(−4.774, 2.637)	0.200	93.410	−2.600		
HWE in controls	−0.175	0.2304	−0.76	0.454	(−0.645, 0.295)	0.001	71.000	−8.060		
GA vs GG										
Region-specified population						0.219	93.930	−4.290	0.800	0.538
European	−1.233	5.004	−0.250	0.807	(−11.500, 9.033)					
North American	2.528	5.217	0.480	0.632	(−8.176, 13.231)					
South American	0.239	5.969	0.040	0.968	(−12.009, 12.487)					
Asian	−1.256	5.629	−0.220	0.825	(−12.807, 10.295)					
Oceanian	referent	referent	referent	referent	referent					
Cancer type						0.241	94.510	−15.000	0.340	0.887
Breast cancer	2.241	3.895	0.580	0.570	(−5.764, 10.247)					
Cervical cancer	−0.400	4.654	−0.090	0.932	(−9.967, 9.167)					
Lung cancer	0.167	6.238	0.030	0.979	(−12.656, 12.990)					
Thyroid cancer	−0.176	4.087	−0.040	0.966	(−8.576, 8.225)					
Other cancer	−0.147	4.086	−0.040	0.972	(−8.545, 8.251)					
Colorectal cancer	referent	referent	referent	referent	referent					
Source of controls	2.226	2.125	1.050	0.305	(−2.160, 6.612)	0.267	94.950	0.030		
Matched controls	−2.547	1.753	−1.450	0.157	(−6.127, 1.033)	0.202	93.760	3.530		
Family history	4.084	1.944	2.100	**0.044**	**(0.113, 8.055)**	0.187	93.750	10.770		
Sample size	−1.178	1.903	−0.620	0.541	(−5.065, 2.709)	0.215	93.760	−2.580		
HWE in controls	−0.206	0.250	−0.820	0.417	(−0.717, 0.305)	0.001	71.820	−9.320		
GG + AA vs GA										
Region-specified population						0.001	68.410	−6.390	0.800	0.534
European	0.659	0.717	0.920	0.366	(−0.813, 2.131)					
North American	0.595	0.736	0.810	0.426	(−0.915, 2.106)					
South American	0.006	0.816	0.010	0.994	(−1.668, 1.680)					
Asian	0.618	0.781	0.790	0.435	(−0.984, 2.221)					
Oceanian	referent	referent	referent	referent	referent					
Cancer type						0.001	69.840	−42.350	0.570	0.719
Breast cancer	0.167	0.407	0.410	0.685	(−0.669, 1.003)					
Cervical cancer	0.500	0.500	1.000	0.327	(−0.529, 1.529)					
Lung cancer	−0.065	0.615	−0.110	0.917	(−1.329, 1.199)					
Thyroid cancer	0.458	0.428	1.070	0.294	(−0.422, 1.338)					
Other cancer	0.193	0.418	0.460	0.648	(−0.666, 1.052)					
Colorectal cancer	referent	referent	referent	referent	referent					
Source of controls	−0.028	0.240	−0.120	0.907	(−0.523, 0.467)	0.002	73.660	−13.140		
Matched controls	0.130	0.186	0.700	0.491	(−0.250, 0.509)	0.001	67.720	−21.020		
Family history	−0.214	0.221	−0.970	0.340	(−0.666, 0.237)	0.001	67.750	−18.530		
Sample size	−0.086	0.184	−0.470	0.643	(−0.463, 0.290)	0.001	67.700	−15.790		
HWE in controls	0.055	0.263	0.210	0.836	(−0.484, 0.594)	0.001	68.690	−17.640		

*P*_J_: *P* value of the joint test for all variables

Significant 95% *CI*s and *P* values were in bold

### Publication bias

**Fig. 2:**
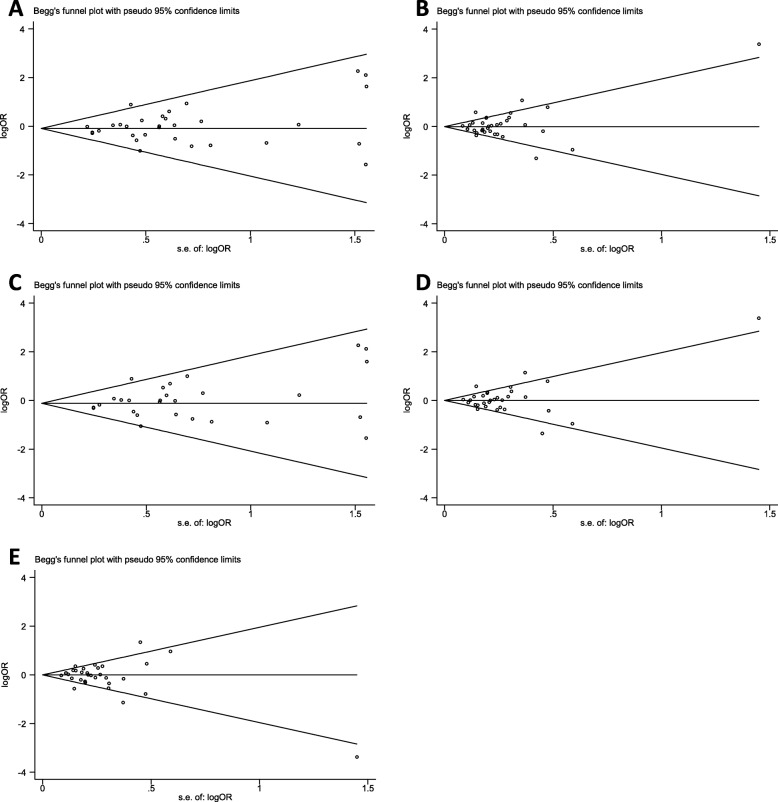
Funnel plots for publication bias of the meta-analysis on rs1801516 polymorphism and overall cancer risk. **a** recessive model: AA vs GG + GA; **b** dominant model: AA+GA vs GG; **c** codominant model: AA vs GG; **d** codominant model: GA vs GG; **e** overdominant model: AA+GG vs GA

Begg’s funnel plot and Egger’s linear regression test were used to assess the publication bias of studies in our meta-analysis. The shape of the funnel plots under four models seemed symmetrical (Fig. 2), and the results of Egger’s test revealed no evidence of significant publication bias (AA vs GG + GA: *P* = 0.266; AA+GA vs GG: *P* = 0.505; AA vs GG: *P* = 0.201; GA vs GG: *P* = 0.574; GG + AA vs GA: *P* = 0.587).

### Sensitivity analyses

**Fig. 3:**
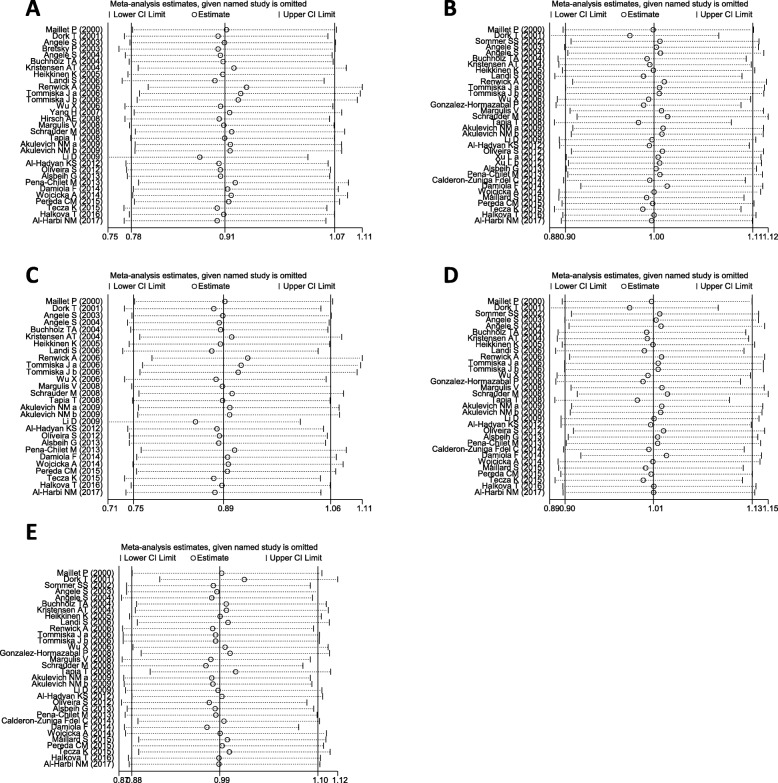
Sensitivity analyses of the studies. **a** recessive model: AA vs GG + GA; **b** dominant model: AA+GA vs GG; **c** codominant model: AA vs GG; **d** codominant model: GA vs GG; **e** overdominant model: AA+GG vs GA

We performed sensitivity analysis by excluding one study at each time to evaluate the influence of each individual study on the overall *OR*s and 95% *CI*s. The results showed that the pooled *OR*s and 95% *CI*s under any model of inheritance were not substantially altered after omitting any individual study (Fig. 3), suggesting that the results of our meta-analysis are credible.

### Trial sequential analysis

**Fig. 4:**
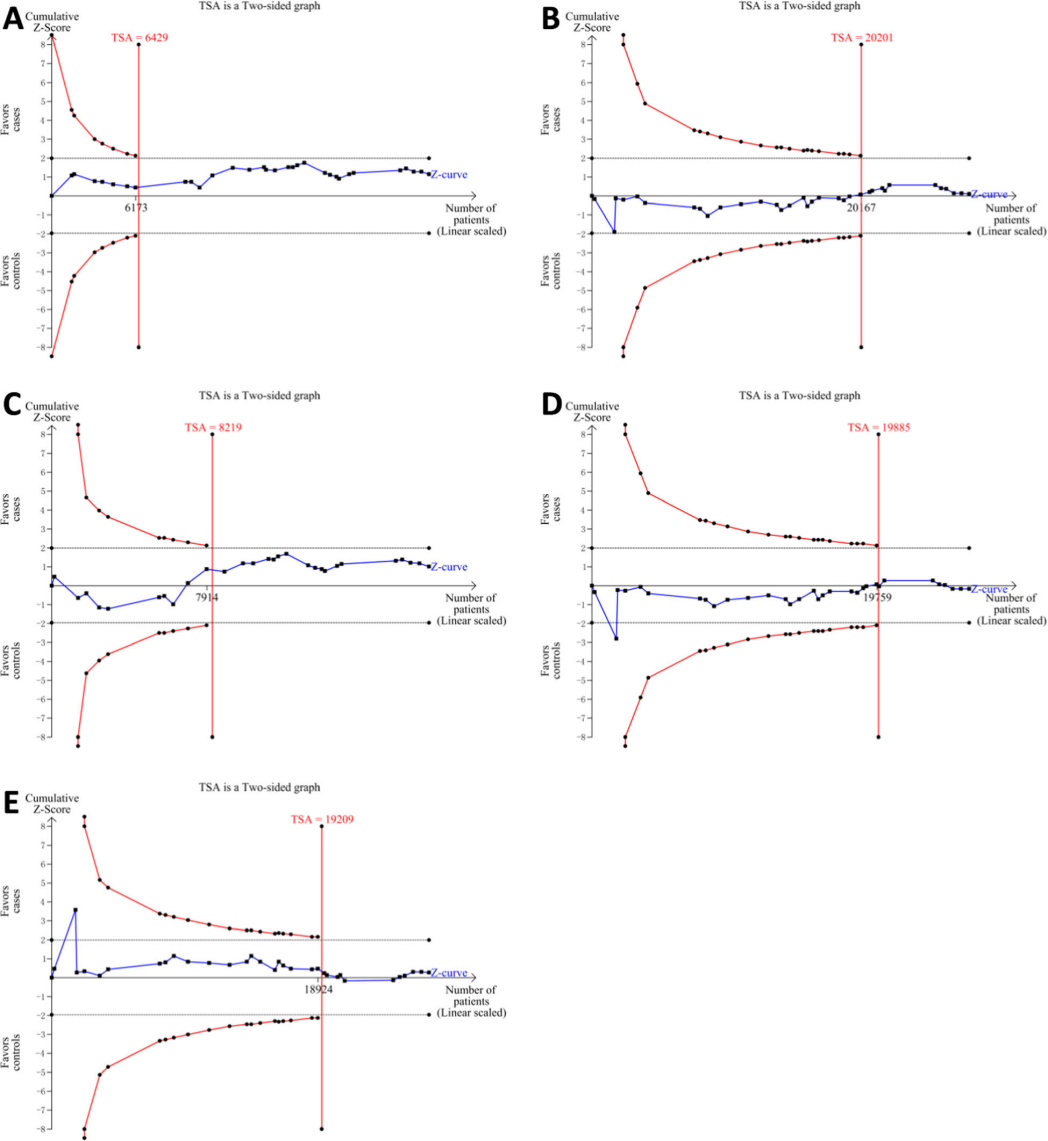
Trial sequential analysis of the association between rs1801516 polymorphism and overall cancer risk. The required information size was calculated based on a two side *α* = 5%, *β* = 25% (power 80%), and a relative risk reduction of 20%. **a** recessive model: AA vs GG + GA; **b** dominant model: AA+GA vs GG; **c** codominant model: AA vs GG; **d** codominant model: GA vs GG; **e** overdominant model: AA+GG vs GA

The results of TSA under four models (five comparisons) are shown in Fig. 4, and they were consistent with the results of the conventional meta-analyses. The blue lines of cumulative *Z*-score didn’t cross the trial sequential monitoring boundaries (red lines sloping inwards), suggesting there is no significant association between rs1801516 polymorphism and cancer risk. Moreover, sample sizes in our overall meta-analyses were all more than the required information sizes (AA vs GG + GA: 6429; AA+GA vs GG: 20201; AA vs GG: 8219; GA vs GG: 19885; GG + AA vs GA: 19209), suggesting that the results of our meta-analyses are reliable.

## Discussion

Studies of rs1801516 polymorphism on cancer risk have been performed for more than ten cancers in previous studies, and breast cancer and thyroid cancer are the two most studied ones. So far, three meta-analyses have been performed on association between rs1801516 polymorphism and breast cancer risk [11, 12, 14], and two meta-analysis have been performed on the association between rs1801516 polymorphism and thyroid cancer risk [10, 55]. Moreover, one meta-analysis focused on this polymorphism and cancer risk despite of cancer types, but it was stratified by the status of radiation exposure [13]. In our meta-analysis, we assessed the association between rs1801516 polymorphism and overall cancer risk for the first time. We found that no significant association existed under any model of inheritance in the overall analysis. Our result was consistent with the finding of the previous study on rs1801516 polymorphism and cancer risk in population without radiation exposure [13]. Therefore, rs1801516 polymorphism may be not associated with overall cancer risk.

In subgroup analyses by region-specified population, cancer types, and family history, significant associations were found for European, South American, Asian, breast cancer, and those with family history. Firstly, results of subgroup analysis by region-specified population were interesting. In European and Asian, reversed results were observed for AA vs GG + GA and AA vs GG. The homozygote AA showed a protective effect against cancer in European, but it presented a susceptible effect for cancer in Asian. Therefore, rs1801516 polymorphism may exert inversed effect on European and Asian. In South American, the other three models (AA+GA vs GG, GA vs GG, and GG + AA vs GA) were significant. Susceptible effect for cancer was observed for AA+GA vs GG and GA vs GG, and protective effect against cancer was observed for GG + AA vs GA. We infer that the results in South American may be attributed to the heterozygote GA, which may be a risk genotype of cancer in South American. Populations from different region may be ethnically different, and this difference may in turn have an influence on cancer susceptibility. Studies have revealed cancer trends differed from ethnicity [56–58], and patients of different ethnicity presents different cancer phenotypes [59, 60]. Besides, discrepancy in distribution of rs1801516 genotype may exist in different populations. Secondly, subgroup analysis by cancer types in our study indicated that AA homozygotes have a relative low risk of breast cancer compared with GG carriers. Three previous meta-analyses [11, 12, 14] have been performed on association between rs1801516 polymorphism and breast cancer risk, and the result for AA vs GG + GA in study of Lu PH et al. [14] is significant, indicating that AA is a low risk genotype. Our results were consistent with those of Lu et al.. Moreover, 13 studies were included in our meta-analysis of breast cancer, which was much more than that in studies of Mao C et al.(eight studies included) [11], Gao LB et al. (nine studies included) [12], and Lu PH et al. (five studies included) [14]. Thus, compared with GG genotype, AA genotype of rs1801516 may be a potential protective factor of breast cancer. Thirdly, for those with family history, AA homozygotes presented low susceptibility of cancer in our meta-analysis. Impact of family history on cancer occurrence and clinical features has been found in different types of cancer, and family history may also exert an influence on cancer through interaction with gene polymorphism [61–63]. In Mao et al.’s meta-analysis [11], subgroup analysis was also performed by family history, but their results are not similar to ours. Difference in sample size between the two meta-analyses of ours and Mao et al.’s may result in the inconsistence of results.

In this present meta-analysis, heterogeneity was observed in models of AA+GA vs GG, GA vs GG, and GG + AA vs GA. To find the source of among-studies’ heterogeneity, we performed meta-regression analysis by region-specified population, cancer type, source of controls, matched controls or not, family history, sample size, and HWE in controls. As a result, family history was a source of heterogeneity for AA+GA vs GG and GA vs GG models. However, for GG + AA vs GA, none of the analyzed factors was detected as a source of heterogeneity. Lifestyle may be the source of heterogeneity. Lifestyle of the subjects, including smoking and alcohol consumption, influences on their susceptibility to cancer [64–66]. However, the 37 studies included in our meta-analysis do not provide adequate information on lifestyle. Moreover, genotyping methods of the included studies are various: more than ten methods used in all the included studies, and multiple methods were used in several individual studies. Diversity of genotyping methods may also be a reason of heterogeneity, but because of diverse methods, we do not put genotyping methods into the analysis of meta-regression. In addition, matching criteria of the included studies with controls matched to cases are different, giving rise to the heterogeneity possibly.

Meta-analysis may report false positive results for the risk of type I error, and such results are commonly attributed to publication bias, heterogeneity among studies, and low quality of the studies. However, a limited number of trials may not give enough information size, thereby leading to a false estimation [67]. In order to comprehensively evaluate the impact of *ATM* rs1801516 polymorphism on cancer risk, we performed TSA to reduce the risk of type I error and to estimate whether further studies are required by calculating the required information size. Sample size in our meta-analysis was more than the required information size, indicating that the results of our meta-analyses are reliable and sufficient to draw a conclusion.

We must admit that there are some limitations in our meta-analysis. Firstly, because of the difference in data presentation of age between studies (mean age, median age, and age group), we didn’t assess the risk stratified by age. Secondly, environmental factors and life style information were not available for all studies, thus effects of these variables were not taken into consideration. Thirdly, year of data collection may also have an effect on heterogeneity, but not all studies in our meta-analysis provide this information, thus year of data collection was not analyzed in our meta-analysis. Fourthly, 12 types of cancer were included in our meta-analysis. However, only one or two studies were performed on the cancers except breast cancer, thyroid cancer, and cervical cancer, and this may potentially make the result biased.

## Conclusions

In summary, *ATM* rs1801516 polymorphism is not associated with overall cancer risk in total population. However, for subgroup analyses, rs1801516 polymorphism is especially associated with breast cancer risk; in addition, this polymorphism is associated with overall cancer risk in Europeans, South Americans, Asians, and those with family history. Owing to the limitations mentioned above, our results should be interpreted with caution.

## Additional files

Additional file 1: **Table S1.** Matching criteria and genotyping method of the eligible studies included in the meta-analysis. (DOCX 24 kb)

## Abbreviations

A-T: Ataxia-telangiectasia
ATM: Ataxia telangiectasia mutated
CI: Confidence interval
DSB: Double-strand break
GWAS: Genome-wide association studies
HBC: Hospital-based controls
HWE: Hardy-Weinberg equilibrium
IR: Ionizing radiation
MAF: Minor allele frequency
NOS: Newcastle-Ottawa Scale
OR: Odds ratio
PBC: Population-based controls
PCR-RFLP: PCR-restriction fragment length polymorphism
PI3-K: Phosphoinositide 3-kinase
PRISMA: Preferred Reporting Items for Systematic Reviews and Meta-Analyses
PTC: Papillary thyroid cancer
RT-PCR: Real time polymerase chain reaction
SNP: Single nucleotide polymorphism
TaqMan: TaqManSNP
