# Supplementary material for: Association between ATM rs1801516 polymorphism and cancer susceptibility: a meta-analysis involving 12,879 cases and 18,054 controls
Source: BMC Cancer. 2018 Nov 1;18:1060. doi: 10.1186/s12885-018-4941-1 (PMC6211574; doi:10.1186/s12885-018-4941-1)
Supplement: Supplementary file 1 — Table S1. Matching criteria and genotyping method of the eligible studies included in the meta-analysis. (DOCX 24 kb) [file 12885_2018_4941_MOESM1_ESM.docx]

| Table S1. Matching criteria and genotyping method of the eligible studies included in the meta-analysis | | | |
| --- | --- | --- | --- |
| First author | Year | Matching criteria | Genotyping method |
|  |  |  |  |
| Maillet P [1] | 2000 | Random | Direct sequencing |
| Dork T [2] | 2001 | Geographic region | SSCP |
| Sommer SS [3] | 2002 | Ethnicity | SSCP |
| Angele S [4] | 2003 | living area | RFLP, SOMA, Sequencing, DHPLC |
| Bretsky P [5] | 2003 | Age | TaqMan |
| Angele S [6] | 2004 | Random | DHPLC |
| Buchholz TA [7] | 2004 | Random | ASO |
| Kristensen AT [8] | 2004 | Random | PCR |
| Heikkinen K [9] | 2005 | Age, geographical region | CSGE |
| Landi S [10] | 2006 | Age, sex, center, referral (or residence) area | Microarray |
| Renwick A [11] | 2006 | Random | CSGE |
| Tommiska J ^a^ [12] | 2006 | Geographical region | Minisequencing, Amplifluor fluorescent genotyping, RFLP |
| Tommiska J ^b^ [12] | 2006 | Geographical region | Minisequencing, Amplifluor fluorescent genotyping, RFLP |
| Wu X [13] | 2006 | Age, gender, ethnicity | TaqMan |
| Yang H [14] | 2007 | Age, gender, ethnicity, smoking status | TaqMan |
| Gonzalez-Hormazabal P [15] | 2008 | Age, ethnic background, geographical area, socioeconomic strata | PCR-RFLP |
| Hirsch AE [16] | 2008 | Age | DHPLC |
| Margulis V [17] | 2008 | Age, sex, ethnicity, county of residence | Taqman, RT-PCR |
| Schrauder M [18] | 2008 | Age, geographical area | RT-PCR |
| Tapia T [19] | 2008 | Random | PCR |
| Akulevich NM ^a^ [20] | 2009 | Age, settlement | PCR-RFLP |
| Akulevich NM ^b^ [20] | 2009 | Age, settlement | PCR-RFLP |
| Li D [21] | 2009 | Age, sex, race. | TaqMan |
| Oliveira S [22] | 2011 | Random | TaqMan |
| Al-Hadyan KS [23] | 2012 | Random | Direct sequencing |
| Xu L ^a^ [24] | 2012 | Random | PCR-RFLP, TaqMan |
| Xu L ^b^ [24] | 2012 | genotypes, Age, lifetime occupational history, smoking history, general health conditions, previous diseases | PCR-RFLP, TaqMan |
| Alsbeih G [25] | 2013 | Age, sex | Direct sequencing |
| Pena-Chilet M [26] | 2013 | Ethnicity | KASPar |
| Calderon-Zuniga Fdel C [27] | 2014 | Random | PCR-RFLP |
| Damiola F [28] | 2014 | Age, sex, settlement | PCR, TaqMan |
| Wojcicka A [29] | 2014 | Random | iPLEX Gold |
| Maillard S [30] | 2015 | Date of birth, gender | HRM |
| Pereda CM [31] | 2015 | Age, sex | HRM |
| Tecza K [32] | 2015 | Age | ASA-PCR, Multiplex-PCR, RFLP-PCR |
| Halkova T [33] | 2016 | Random | TaqMan |
| Al-Harbi NM [34] | 2017 | Age, sex | Direct sequencing |
| PCR, polymerase chain reaction; RFLP, restriction fragment length polymorphism analysis; DHPLC, highperformance liquid chromatography; ASO, allele-specific oligonucleotide assay; HRM, High-Resolution Melting curve; ASA-PCR, allele-specific amplification PCR; PCR-RFLP, PCR-restriction fragment length polymorphism assay; RT-PCR, real time PCR; SSCP, single strand conformation polymorphism; CSGE, conformation-sensitive gel electrophoresis; SOMA, short oligonucleotide mass analysis | | | |
| ^a,b^ Two independent case-control studies were presented for the same original study | | | |

**References**

1. Maillet P. Chappuis PO. Vaudan G. Dobbie Z. Muller H. Hutter P, et al. A polymorphism in the ATM gene modulates the penetrance of hereditary non-polyposis colorectal cancer. Int J Cancer. 2000;88(6): 928-31.

2. Dork T, Bendix R, Bremer M, Rades D, Klopper K, Nicke M, et al. Spectrum of ATM gene mutations in a hospital-based series of unselected breast cancer patients. Cancer Res. 2001;61(20): 7608-15.

3. Sommer SS, Buzin CH, Jung M, Zheng J, Liu Q, Jeong SJ, et al. Elevated frequency of ATM gene missense mutations in breast cancer relative to ethnically matched controls. Cancer Genet Cytogenet. 2002;134(1): 25-32.

4. Angele S, Romestaing P, Moullan N, Vuillaume M, Chapot B, Friesen M, et al. ATM haplotypes and cellular response to DNA damage: association with breast cancer risk and clinical radiosensitivity. Cancer Res. 2003;63(24): 8717-25.

5. Bretsky P, Haiman CA, Gilad S, Yahalom J, Grossman A, Paglin S, et al. The relationship between twenty missense ATM variants and breast cancer risk: The multiethnic cohort. Cancer Epidemiology Biomarkers & Prevention. 2003;12(8): 733-38.

6. Angele S, Falconer A, Edwards SM, Dork T, Bremer M, Moullan N, et al. ATM polymorphisms as risk factors for prostate cancer development. British Journal of Cancer. 2004;91(4): 783-87.

7. Buchholz TA, Weil MM, Ashorn CL, Strom EA, Sigurdson A, Bondy M, et al. A Ser49Cys variant in the ataxia telangiectasia, mutated, gene that is more common in patients with breast carcinoma compared with population controls. Cancer. 2004;100(7): 1345-51.

8. Kristensen AT, Bjorheim J, Wiig J, Giercksky KE, Ekstrom PO. DNA variants in the ATM gene are not associated with sporadic rectal cancer in a Norwegian population-based study. Int J Colorectal Dis. 2004;19(1): 49-54.

9. Heikkinen K, Rapakko K, Karppinen SM, Erkko H, Nieminen P, Winqvist R. Association of common ATM polymorphism with bilateral breast cancer. Int J Cancer. 2005;116(1): 69-72.

10. Landi S, Gemignani F, Canzian F, Gaborieau V, Barale R, Landi D, et al. DNA repair and cell cycle control genes and the risk of young-onset lung cancer. Cancer Res. 2006;66(22): 11062-9.

11. Renwick A, Thompson D, Seal S, Kelly P, Chagtai T, Ahmed M, et al. ATM mutations that cause ataxia-telangiectasia are breast cancer susceptibility alleles. Nature Genetics. 2006;38(8): 873-75.

12. Tommiska J, Jansen L, Kilpivaara O, Edvardsen H, Kristensen V, Tamminen A, et al. ATM variants and cancer risk in breast cancer patients from Southern Finland. BMC Cancer. 2006;6: 209.

13. Wu X, Gu J, Grossman HB, Amos CI, Etzel C, Huang M, et al. Bladder cancer predisposition: a multigenic approach to DNA-repair and cell-cycle-control genes. Am J Hum Genet. 2006;78(3): 464-79.

14. Yang H, Spitz MR, Stewart DJ, Lu C, Gorlov IP, Wu X. ATM sequence variants associate with susceptibility to non-small cell lung cancer. Int J Cancer. 2007;121(10): 2254-9.

15. Gonzalez-Hormazabal P, Bravo T, Blanco R, Valenzuela CY, Gomez F, Waugh E, et al. Association of common ATM variants with familial breast cancer in a South American population. BMC Cancer. 2008;8: 117.

16. Hirsch AE, Atencio DP, Rosenstein BS. Screening for ATM sequence alterations in African-American women diagnosed with breast cancer. Breast Cancer Res Treat. 2008;107(1): 139-44.

17. Margulis V, Lin J, Yang H, Wang W, Wood CG, Wu X. Genetic susceptibility to renal cell carcinoma: the role of DNA double-strand break repair pathway. Cancer Epidemiol Biomarkers Prev. 2008;17(9): 2366-73.

18. Schrauder M, Frank S, Strissel PL, Lux MP, Bani MR, Rauh C, et al. Single nucleotide polymorphism D1853N of the ATM gene may alter the risk for breast cancer. Journal of Cancer Research and Clinical Oncology. 2008;134(8): 873-82.

19. Tapia T, Sanchez A, Vallejos M, Alvarez C, Moraga M, Smalley S, et al. ATM allelic variants associated to hereditary breast cancer in 94 Chilean women: susceptibility or ethnic influences? Breast Cancer Res Treat. 2008;107(2): 281-8.

20. Akulevich NM, Saenko VA, Rogounovitch TI, Drozd VM, Lushnikov EF, Ivanov VK, et al. Polymorphisms of DNA damage response genes in radiation-related and sporadic papillary thyroid carcinoma. Endocr Relat Cancer. 2009;16(2): 491-503.

21. Li D, Suzuki H, Liu B, Morris J, Liu J, Okazaki T, et al. DNA repair gene polymorphisms and risk of pancreatic cancer. Clin Cancer Res. 2009;15(2): 740-6.

22. Oliveira S, Ribeiro J, Sousa H, Pinto D, Baldaque I, Medeiros R. Genetic polymorphisms and cervical cancer development: ATM G5557A and p53bp1 C1236G. Oncol Rep. 2012;27(4): 1188-92.

23. Al-Hadyan KS, Al-Harbi NM, Al-Qahtani SS, Alsbeih GA. Involvement of single-nucleotide polymorphisms in predisposition to head and neck cancer in Saudi Arabia. Genet Test Mol Biomarkers. 2012;16(2): 95-101.

24. Xu L, Morari EC, Wei Q, Sturgis EM, Ward LS. Functional variations in the ATM gene and susceptibility to differentiated thyroid carcinoma. J Clin Endocrinol Metab. 2012;97(6): 1913-21.

25. Alsbeih G, Al-Harbi N, El-Sebaie M, Al-Badawi I. HPV prevalence and genetic predisposition to cervical cancer in Saudi Arabia. Infect Agent Cancer. 2013;8(1): 15.

26. Pena-Chilet M, Blanquer-Maceiras M, Ibarrola-Villava M, Martinez-Cadenas C, Martin-Gonzalez M, Gomez-Fernandez C, et al. Genetic variants in PARP1 (rs3219090) and IRF4 (rs12203592) genes associated with melanoma susceptibility in a Spanish population. BMC Cancer. 2013;13: 160.

27. Calderon-Zuniga Fdel C, Ocampo-Gomez G, Lopez-Marquez FC, Recio-Vega R, Serrano-Gallardo LB, Ruiz-Flores P. ATM polymorphisms IVS24-9delT, IVS38-8T>C, and 5557G>A in Mexican women with familial and/or early-onset breast cancer. Salud Publica Mex. 2014;56(2): 206-12.

28. Damiola F, Byrnes G, Moissonnier M, Pertesi M, Deltour I, Fillon A, et al. Contribution of ATM and FOXE1 (TTF2) to risk of papillary thyroid carcinoma in Belarusian children exposed to radiation. Int J Cancer. 2014;134(7): 1659-68.

29. Wojcicka A, Czetwertynska M, Swierniak M, Dlugosinska J, Maciag M, Czajka A, et al. Variants in the ATM-CHEK2-BRCA1 axis determine genetic predisposition and clinical presentation of papillary thyroid carcinoma. Genes Chromosomes Cancer. 2014;53(6): 516-23.

30. Maillard S, Damiola F, Clero E, Pertesi M, Robinot N, Rachedi F, et al. Common variants at 9q22.33, 14q13.3, and ATM loci, and risk of differentiated thyroid cancer in the French Polynesian population. PLoS One. 2015;10(4): e0123700.

31. Pereda CM, Lesueur F, Pertesi M, Robinot N, Lence-Anta JJ, Turcios S, et al. Common variants at the 9q22.33, 14q13.3 and ATM loci, and risk of differentiated thyroid cancer in the Cuban population. Bmc Genetics. 2015;16.

32. Tecza K, Pamula-Pilat J, Kolosza Z, Radlak N, Grzybowska E. Genetic polymorphisms and gene-dosage effect in ovarian cancer risk and response to paclitaxel/cisplatin chemotherapy. J Exp Clin Cancer Res. 2015;34: 2.

33. Halkova T, Dvorakova S, Sykorova V, Vaclavikova E, Vcelak J, Vlcek P, et al. Polymorphisms in selected DNA repair genes and cell cycle regulating genes involved in the risk of papillary thyroid carcinoma. Cancer Biomark. 2016;17(1): 97-106.

34. Al-Harbi NM, Bin Judia SS, Mishra KN, Shoukri MM, Alsbeih GA. Genetic Predisposition to Cervical Cancer and the Association With XRCC1 and TGFB1 Polymorphisms. Int J Gynecol Cancer. 2017;27(9): 1949-56.
